# Impact of Different Drying Methods on the Microbiota, Volatilome, Color, and Sensory Traits of Sea Fennel (*Crithmum maritimum* L.) Leaves

**DOI:** 10.3390/molecules28207207

**Published:** 2023-10-21

**Authors:** Antonietta Maoloni, Federica Cardinali, Vesna Milanović, Anna Reale, Floriana Boscaino, Tiziana Di Renzo, Ilario Ferrocino, Giorgia Rampanti, Cristiana Garofalo, Andrea Osimani, Lucia Aquilanti

**Affiliations:** 1 Dipartimento di Scienze Agrarie, Alimentari e Ambientali, Università Politecnica delle Marche, 60131 Ancona, Italy; a.maoloni@univpm.it (A.M.); f.cardinali@univpm.it (F.C.); v.milanovic@univpm.it (V.M.); g.rampanti@pm.univpm.it (G.R.); c.garofalo@univpm.it (C.G.); a.osimani@univpm.it (A.O.); 2 Istituto di Scienze dell’Alimentazione (ISA), Consiglio Nazionale delle Ricerche (CNR), 83100 Avellino, Italy; anna.reale@isa.cnr.it (A.R.); floriana.boscaino@isa.cnr.it (F.B.); tiziana.direnzo@isa.cnr.it (T.D.R.); 3 Department of Agricultural, Forest, and Food Science, University of Turin, Largo Paolo Braccini 2, Grugliasco, 10095 Turin, Italy; ilario.ferrocino@unito.it

**Keywords:** rock samphire, dried spices, microwave drying, metataxonomic, *Bacillus*, *Pseudomonas*, *Alternaria*, *Cryptococcus*

## Abstract

Sea fennel (*Crithmum maritimum* L.) is a strongly aromatic herb of the Apiaceae family, whose full exploitation by the modern food industry is of growing interest. This study aimed at investigating the microbiological quality, volatile profile, and sensory traits of sea fennel spices produced using room-temperature drying, oven drying, microwave drying, and freeze drying. All the assayed methods were able to remove moisture up until water activity values below 0.6 were reached; however, except for microwave drying, none of the assayed methods were effective in reducing the loads of contaminating microorganisms. The metataxonomic analysis highlighted the presence of phytopathogens and even human pathogens, including members of the genera *Bacillus*, *Pseudomonas*, *Alternaria,* and *Cryptococcus*. When compared to fresh leaves, dried leaves showed increased L* (lightness) and c* (chroma, saturation) values and reduced hue angle. Dried leaves were also characterized by decreased levels of terpene hydrocarbons and increased levels of aldehydes, alcohols, and esters. For the sensory test, the microwave-dried samples obtained the highest appreciation by the trained panel. Overall, the collected data indicated microwave drying as the best option for producing sea fennel spices with low microbial loads, brilliant green color, and high-quality sensory traits.

## 1. Introduction

According to the International Organization for Standardization (ISO), spices are defined as “*vegetable products or mixtures thereof, free from extraneous matter, used for flavoring, seasoning, and imparting aroma in foods*”. Fresh spices are highly perishable products due to their high moisture content. Dehydration is undoubtedly one of the oldest techniques used for preservation of herbs and spices since it extends their shelf life by reducing the moisture content and, hence, water activity (a_w_), thus leading to the inhibition of microorganisms [1,2].

To date, different drying methods have been assayed for the dehydration of herbs and spices, including convective air drying, microwave drying, and freeze drying. Convective air drying involves exposing fresh herbs and spices to a flow of hot air, which removes moisture from the surface of the product by means of evaporation. The process involves two transport mechanisms that occur simultaneously and in opposite directions: a flow of heat from the hot air to the surface and, hence, to the interior of the product, and a flow of mass (steam) from the inner part of the product to the surface and, hence, to the air [3,4]. Convective air drying is widely used in the food industry due to the low cost of this technology [3]. In microwave drying, heat is generated within the product due to the absorption of microwave energy by water molecules. Subsequently, water evaporation occurs within the product, followed by water diffusion as steam moves from the interior of the product to its surface. The capability of this method for producing high-quality dried herbs within a short processing time is widely acknowledged [3,5,6]. Nevertheless, further research efforts are needed for optimizing the process parameters for drying of species with different microwave energy absorption capacities [7]. Lastly, the freeze-drying technology removes moisture through the sublimation of water from the solid to the vapor state. Despite the high cost, this method is widely used for drying fruits and vegetables, whose bioactive compounds can be retained due to the use of low temperatures and the lack of water and oxygen during the process [8].

Besides their culinary use as flavoring agents, some spices, such as basil, thyme, coriander, cumin, oregano, and parsley, are used for medicinal purpose in the traditional Mediterranean diet due to their high concentration of bioactive compounds, mainly consisting of polyphenols and essential oils [9,10,11,12].

Although the antimicrobial properties of some of these spices have been identified, their exploitation for enhancing the savory experience of foods might also lead to microbial contamination and spoilage. Endospores produced by spore formers introduced by spices into foods, mainly belonging to the genera *Bacillus* and *Clostridium*, can survive through different preparation processes, including thermal treatments, thus leading to changes in food quality and safety during storage [13].

*Crithmum maritimum* L., commonly known as sea fennel, is a highly aromatic herb belonging to the Apiaceae family, like parsley, coriander, and cumin [14,15,16]. Traditionally, leaves can be consumed fresh in salads for their spicy and salty taste, cooked in soups, or consumed as pickles [17,18,19,20], whereas flower tops and stalks are used for the preparation of herbal teas and infusions [21]. Previous studies have suggested that the production of dried spices from sea fennel can represent an opportunity for the full exploitation of the economic potential of this perennial herb [22,23]. In this regard, a few exploratory studies have evaluated the effect of different drying methods on the organoleptic properties of sea fennel dried spices [24,25,26]. In more detail, drying of sea fennel allowed a very interesting shelf-stable product to be placed on the market [26]. Freeze-dried sea fennel was characterized by herbal and fresh notes, whereas hearty and tobacco notes were detected when hot air drying was applied [24]. Freeze-died as well as microwave-dried leaves better retained the color of fresh sea fennel; by contrast, hot drying leaves resulted in darkening [24,25]. In general, microwave-dried leaves gained a high appreciation when panel tests were conducted [25]. However, to the authors’ best knowledge, the impact of these methods on the volatile profile, microbiota, color, and sensory traits of sea fennel dried spices has not yet been investigated.

Given these premises, this study aimed to investigate the microbiological quality, volatile profile, color, and sensory traits of samples of dried sea fennel spices produced via drying methods most frequently applied to herbs and vegetable material, namely room-temperature drying, oven drying, microwave drying, and freeze drying. To this end, aliquots of the resulting leaves were subjected to culture-dependent assays for the enumeration of target microbial groups (e.g., total mesophilic aerobes, Enterobacteriaceae, yeasts, and molds) as well as metagenomic analysis for the disclosure of the bacterial and fungal communities. In parallel, for each drying method, weight loss (%), water activity (a_w_), volatile organic compounds (VOCs), color, and sensory attributes were determined.

## 2. Results

### 2.1. Drying Parameters

The drying parameters, weight loss, and final a_w_ mean values are reported in Table 1.

The moisture content of chopped fresh sea fennel leaves was significantly higher in batch 1 (85.89 ± 0.20% wb) than batch 2 (83.21 ± 0.35% wb), whereas no significant differences were observed between the two batches for the a_w_ mean values, which corresponded to values of 1.00 ± 0.01 and 0.99 ± 0.01 for batch 1 and batch 2, respectively.

With respect to the duration of the drying process for weight stabilization, MD was a faster drying method, followed by FD, OD, and RTD, respectively. However, all the assayed methods produced a weight loss (%) corresponding to the moisture content of the two batches of fresh sea fennel. Regarding a_w_, higher mean values were generally found in the samples dried at room temperature in respect of the other three methods; in batch 2, comparable a_w_ values were recorded for both microwaved and room-temperature-dried sea fennel.

### 2.2. Viable Plate Counts

The results of viable plate counting are reported in Table 2. As a general trend, mesophilic aerobic bacteria, spore formers, and Enterobacteriaceae occurred at comparable or even higher levels in the dried sea fennel samples than in fresh sea fennel samples, irrespective of the drying method used. The only exception was the sea fennel samples subjected to MD, which were characterized by significantly lower levels of mesophilic aerobic bacteria and Enterobacteriaceae than fresh sea fennel. In more detail, MD resulted in a strong reduction in Enterobacteriaceae, yeasts, and molds, with the final loads below the detection limit (1.0 Log CFU g^−1^). Yeasts and molds exhibited different trends depending on the drying method and the batch. Overall, a significantly lower yeast load was observed in oven-dried samples than in fresh sea fennel.

### 2.3. Metataxonomic Analysis

**Figure 1:**
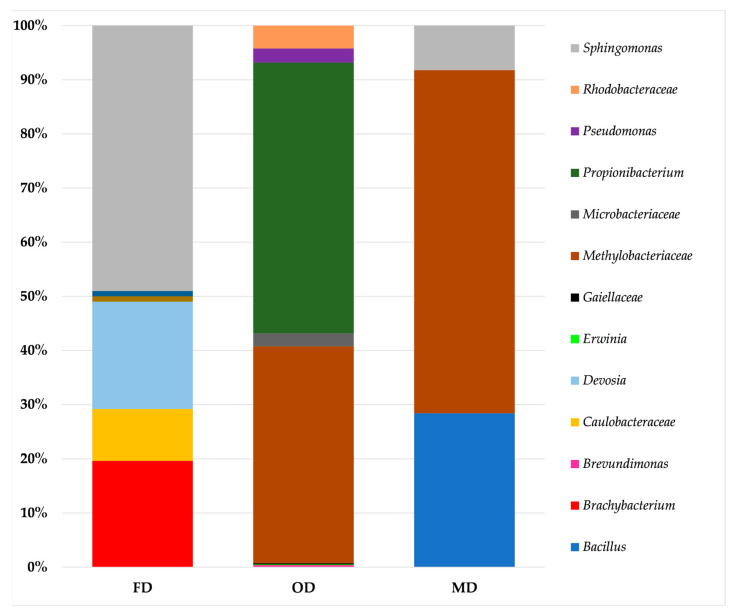
Relative frequency of bacterial amplicon sequencing variants (ASVs) detected in the samples of dried sea fennel spices. For an explanation of the drying methods and batches used, see Table 1.

**Figure 2:**
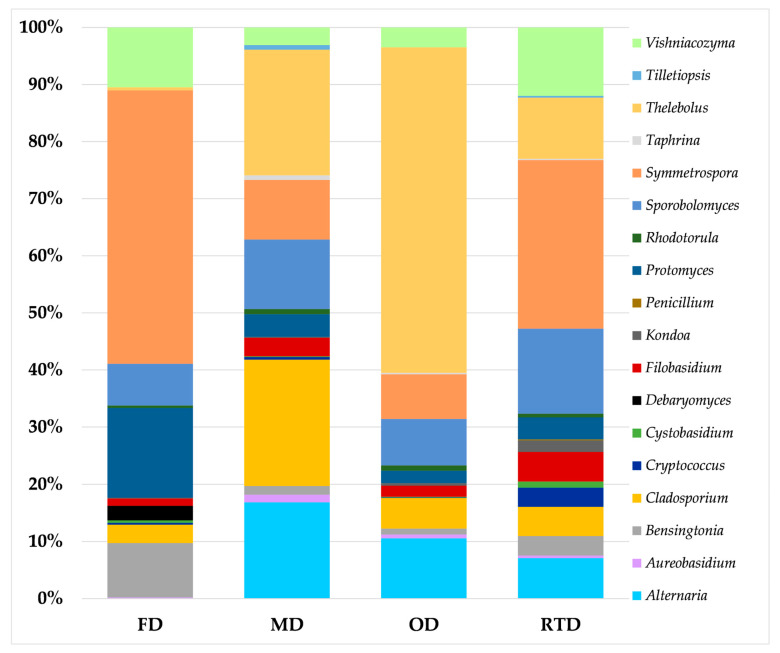
Relative frequency of fungal amplicon sequencing variants (ASVs) detected in the samples of dried sea fennel spices. For an explanation of the drying methods and batches used, see Table 1.

The results of the metataxonomic analysis of the bacterial and fungal biota of the samples of dried spices are reported in Figure 1 and Figure 2, respectively. For bacteria, twelve taxa were identified overall, including seven genera (*Bacillus*, *Brachybacterium*, *Devosia*, *Erwinia*, *Pseudomonas*, *Propionibacterium*, and S*phingomonas*) and five families, namely Caulobacteraceae, Gaiellaceae, Microbacteriaceae, Rhodobacteraceae, and Methylobacteriaceae (Figure 1, Table S1), with the last among these families dominating in both oven- and microwave-dried spices.

The same family also prevailed in fresh sea fennel leaves, whose analysis was aimed at optimizing the DNA extraction and amplification procedures. For fungi, a core of 13 genera was stably detected in the samples, irrespective of the drying method applied; these included *Aureobasidium*, *Bensingtonia*, *Cladosporium*, *Cryptococcus*, *Cystobasidium*, *Filobasidium*, *Kondoa*, *Protomyces*, *Rhodotorula*, *Sporobolomyces*, *Symmetrospora*, *Thelebolus*, and *Vishniacozyma*. Two additional genera, *Alternaria* and *Taphrina*, were also found in all samples except for the freeze-dried samples, whereas the genus *Tilletiopsis* was detected in the samples subjected to room-temperature drying and microwave drying. Furthermore, the genera *Debaryomyces* and *Penicillium* were only found in the freeze-dried and room-temperature-dried samples, respectively (Figure 2, Table S2). The first two genera were also prevalent in fresh sea fennel leaves.

### 2.4. Color

The results of the color assessment are reported in Table 3.

As a general trend, for almost all CIELab coordinates except for L (lightness) and all drying methods, a high variability was observed between the two batches. Regarding L, the lowest values were found for fresh sea fennel leaves (used a control), whereas the highest values for the same coordinate were recorded in the freeze-dried samples.

For the a* coordinate (red (+)/green (−) axis), significantly lower negative values were exhibited by fresh and oven-dried leaves, whereas the freeze-dried samples showed higher negative values. Regarding the b* coordinate (axis yellow (+)/blue (−)), the lowest values were measured in fresh sea fennel, followed by oven-dried leaves, which showed slightly higher values.

**Figure 3:**
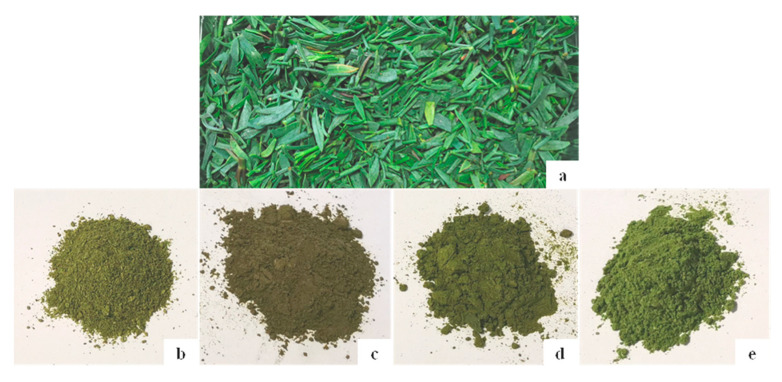
Fresh sea fennel leaves (**a**) and samples of dried sea fennel spices obtained via room-temperature drying (**b**); oven drying (**c**); microwave drying (**d**); and freeze drying (**e**).

All four drying methods showed a significant decrease in the values of hue angle (h°), with the lowest values recorded in the oven-dried samples, and a significant increase in the values of chroma (C). Images of fresh sea fennel leaves and the four prototypes of dried sea fennel spices are shown in Figure 3.

### 2.5. VOCs

The HS/SPME-GC/MS analysis of fresh and dried sea fennel leaves identified the presence of 53 VOCs (Table 4), which were classified into eight classes: aldehydes (9 compounds), alcohols (3 compounds), esters (6 compounds), acids (1 compound), terpene hydrocarbons (16 compounds), oxygenated terpenes (7 compounds), sesquiterpene hydrocarbons (10 compounds), and phenyl propanoids (1 compound).

Terpene hydrocarbons were the most abundant volatile compounds in all the samples, followed by alcohols and oxygenated terpenes. The other volatile compounds occurred at very low levels. In more detail, terpene hydrocarbons accounted for 99% of the compounds found in fresh sea fennel and 95, 93, 83, and 72% of the compounds found in chopped sea fennel leaves subjected to RTD, MD, FD, and OD, respectively.

Limonene was the most abundant compound, followed by β-terpinene and cymene. Smaller amounts of sabinene, trans-ocimene, and β-myrcene were also found. The different drying treatments resulted in a drastic reduction in terpenes, mainly in the samples subjected to OD and FD.

Oxygenated terpenes and sesquiterpene hydrocarbons were also found in trace amounts. The drying treatments could be assumed to have an impact on the levels of these compounds, resulting in their lower levels, based on the higher amounts generally detected in fresh sea fennel. No esters were detected in the fresh samples or in the microwave-treated samples, while low amounts of esters were found in the samples subjected to RTD, OD, and FD.

Aldehydes and acids were found in trace amounts in all analyzed samples, with acetic acid being the only organic acid detected.

Regarding alcohols, fresh sea fennel was characterized by very low amounts of these compounds, whereas the dried sea fennel samples were characterized by higher amounts. In detail, alcohols represented about 1% of volatile compounds in fresh sea fennel and 4, 6, 15, and 26% of volatile compounds in sea fennel dried via MD, FD, RTD, and OD, respectively. Ethyl alcohol was the most representative alcohol.

Traces of phenyl propanoids (dillapiole) were found in all the analyzed samples.

**Figure 4:**
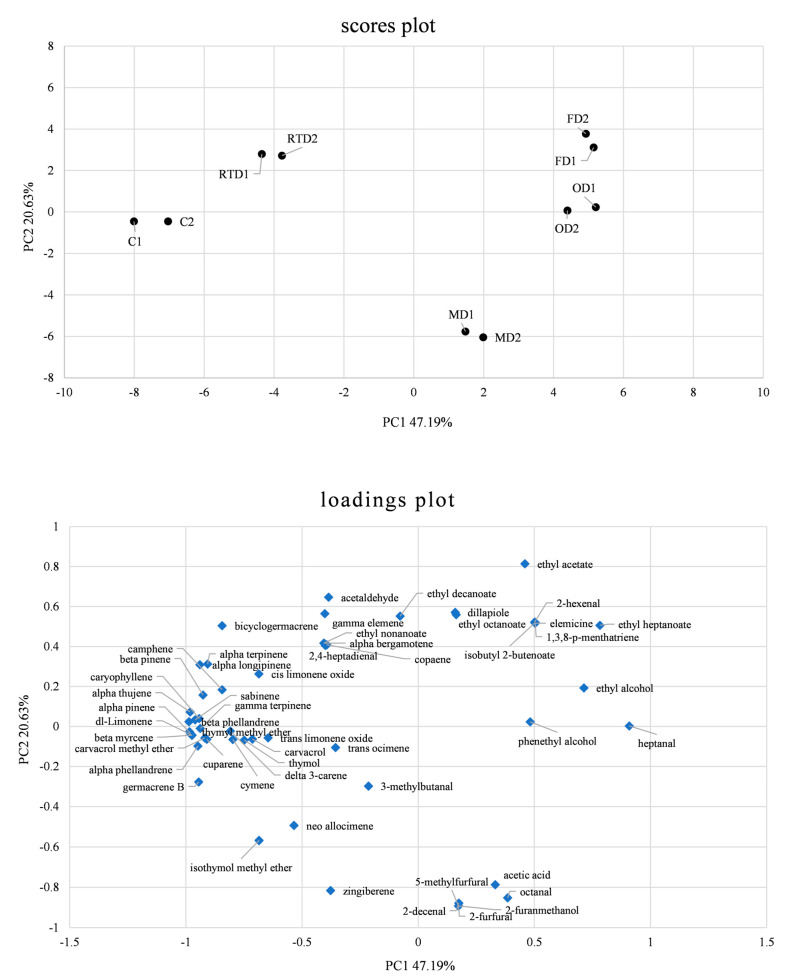
Principal component analysis (PCA) of volatile organic compounds (VOCs) identified in fresh sea fennel (C, control) and in samples of sea fennel spices obtained via room-temperature drying (RTD), oven drying (OD), freeze drying (FD), and microwave drying (MD) in batch 1 (1) and batch 2 (2).

To better understand the differences between the analyzed samples, a PCA of the 53 VOCs detected was carried out, and the results are shown in Figure 4. The two principal components (PCs) explained ca. 67.82% of the total variance of the data. Several compounds loaded positively on PC1, including aldehydes, alcohols, esters, and acids, while numerous other substances, such as terpene hydrocarbons, oxygenated terpenes, and sesquiterpene, loaded negatively on PC1.

Based on the main class of volatile compounds detected, the samples of fresh sea fennel were clearly separated from those dried at room temperature and even more so from those subjected to OD, FD, and MD. The drying process significantly influenced the aroma pattern of the analyzed samples. As shown in the score plot, the analyzed samples are clearly clustered based on their treatment (no drying, FD, RTD, OD, and MD). In fact, according to the first PC (explaining 47.19% of the total variance), the sea fennel samples treated using MD, FD, and OD are distributed opposite to the fresh and room-temperature-dried sea fennel samples. According to the second PC (explaining 20.63% of the total variance), the fresh and microwave-dried sea fennel samples are separated from the other samples. In detail, the undried samples are in the third quadrant, the room-temperature-dried samples are in the second quadrant, the microwave-dried samples are in the fourth quadrant, and the oven-dried and freeze-dried samples are in the first quadrant of the score plot.

The loading plot clearly shows how strongly VOCs influence the clustering of the samples, e.g., high levels of terpenes contribute to the grouping of fresh sea fennel, high levels of furfurals contribute to that of the microwave-dried samples, and high levels of alcohols contribute to that of the oven-dried samples.

### 2.6. Sensory Analysis

**Figure 5:**
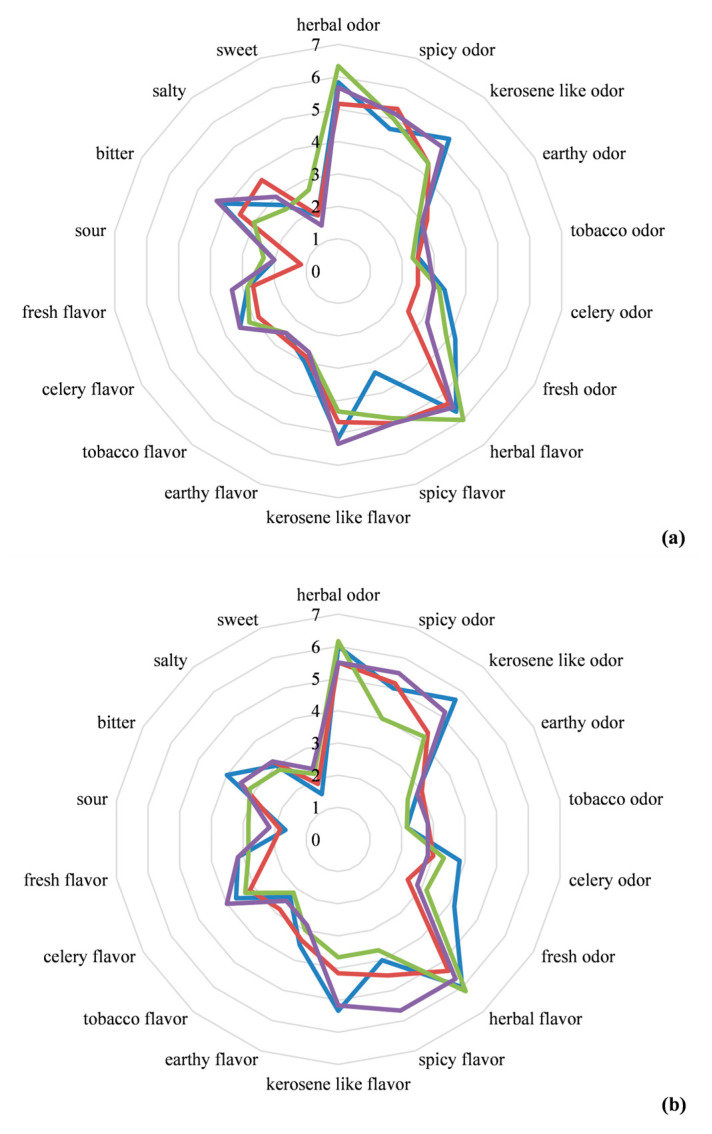
Results of the sensory analysis performed on the two batches (1, panel (**a**); 2, panel (**b**)) of samples of dried sea fennel spices obtained via 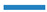 room-temperature drying, 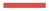 oven drying, 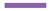 microwave drying, and 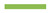 freeze drying. Results are shown as the mean values of three replicates per batch. Each sample was evaluated by a trained panel, consisting of 10 non-smoking tasters aged between 26 and 50, for the presence and intensity of seven odor and flavor descriptors (herbal, spicy, kerosene-like, earthy, tobacco, celery, and fresh) and four taste descriptors (sour, bitter, salty, and sweet). Each descriptor was evaluated by assigning a score ranging between 1 and 9, with 1 expressing the lowest and 9 expressing the highest intensity.

The results of the sensory analysis are depicted in Figure 5.

An almost overlapping picture emerged when comparing the two batches assayed, irrespective of the drying method, with just a few slight variations. Like fresh sea fennel, all samples of dried sea fennel spices were characterized by intense herbaceous and kerosene-like odors and flavors, as well as a “spicy” note. The “earthy” and “tobacco” odors were perceived with the highest intensity in the oven- and microwave-dried samples. By contrast, the lowest intensity for these odors was recorded in the freeze-dried samples. For the “earthy” and “tobacco” flavors, the lowest scores were attributed to the freeze-dried samples. Overall, the freeze-dried samples were characterized by significantly lower scores for bitter and salty taste than either the fresh sea fennel samples or other samples of dried spices.

The microwave-dried samples generally received a higher overall appreciation, with values of 6.7 ± 1.2 and 6.5 ± 0.8 for overall acceptance in terms of liking in batch 1 and 2, respectively.

## 3. Discussion

All four drying methods investigated in this study led to an almost complete removal of moisture from fresh chopped sea fennel leaves, with a reduction in a_w_ below 0.6. Undoubtedly, a_w_ is one of the most important factors affecting microbial growth and toxin production, and its reduction is the main goal of food dehydration [2,27]. More specifically, an a_w_ value of 0.6 conventionally represents the threshold for growth inhibition of both bacteria and eumycetes [28]. Hence, it guarantees the protection of dehydrated foods, including spices, against microbial spoilage during their shelf-life.

As far as microbial viable counts are concerned, the dried samples were characterized by comparable or even higher microbial loads than fresh sea fennel. Exceptions to this trend were the samples subjected to OD at 45 °C, which resulted in a significant reduction in yeasts, and the samples subjected to MD, which caused a significant reduction in all microbial groups, except for spore formers.

For RTD and FD, the trends observed can be easily explained by the mild and low temperatures applied, corresponding to 18–20 °C and <0 °C, respectively, which are unable to exert a microbicidal effect [29,30,31]. For OD, the reduction in the yeast population could again be ascribed to the specific temperature applied [32].

Finally, the reduction in the microbial loads of mesophilic aerobic bacteria, yeasts, and molds in the microwave-dried samples could be explained by the sterilizing capability of microwave treatment, which, in turn, is attributable to a combination of thermal and non-thermal effects [33]. Despite the documented capability of microwave treatment in killing bacterial spores [34,35], some authors reported a higher resistance of bacterial endospores to microwave radiation than vegetative cells [36,37]. This evidence might explain the microbial counts of spore formers observed in the microwave-dried sea fennel samples.

Furthermore, the increase in the microbial loads of some microbial groups might be ascribed to the apparent weight loss registered in all the dried samples.

Spices are foods with low water activity (a_w_ < 0.7), which inhibits microbial growth; however, microorganisms that survive the drying process can remain viable during storage, thus representing a potential hazard when dried spices are added to ready-to-eat or cooked foods [31,38]. In recent years, foodborne outbreaks related to the consumption of dried herbs and spices, mainly caused by *Bacillus cereus*, *Salmonella senftenberg*, and *Clostridium perfringens*, have been reported by the European Food Safety Authority (EFSA) [39]. As suggested by the EFSA, microbial contamination of fresh plants represents one of the main factors affecting the microbiological quality of spices; hence, proper cleaning procedures, including the use of sanitizing solutions, could enable the production of dried spices of high microbiological quality [40]. The metataxonomic analysis performed in this study revealed a higher yeast than bacterial diversity in terms of number of identified taxa. Regarding bacteria, members of the family Methylobacteriaceae prevailed in both the fresh sea fennel and dried samples. Methylobacteriaceae are a large family of *Alphaproteobacteria* within the order *Rhizobiales*, which currently includes three genera, namely *Meganema*, *Microvirga*, and *Methylobacterium* [41]. The last genus currently contains 44 validated species that are ubiquitous in the natural environment, as free-living organisms in water and soil, on the phylloplane, and in plant leaf, stem, and root tissues [41].

Further bacterial families that are generally found in soil were also identified in a few samples of dried sea fennel spices, including Caulobacteraceae [42], Gaiellaceae [43,44], and Microbacteriaceae [45]. For the identified genera, ubiquitous (*Bacillus* spp., *Propionibacterium* spp., and *Pseudomonas* spp.) and environmental (*Brachybacterium* spp.) bacteria as well as soil- (*Devosia* spp.) and plant-associated (*Erwinia* spp., *Sphingomonas* spp.) microorganisms were detected.

Members of the genera *Erwinia*, *Pseudomonas*, and *Bacillus* have previously been isolated from herbs and spices [13,46]. A few of the genera identified in this study are also known to include species involved in the development of human diseases, including *Bacillus* [47], *Propionibacterium* [48], *Sphingomonas* [49], and *Pseudomonas* [50]. Regarding the detection of bacilli ascribed to *Bacillus* spp., most species within this genus are non-pathogenic; however, *Bacillus cereus* is the causative agent of diarrheal and emetic food poisoning syndromes [47]. More specifically, *B. cereus* spores can survive a wide range of cooking temperatures and grow in foods seasoned with herbs and spices at room temperature, thus leading to the exposure of consumers to the risk of food poisoning [13].

Of note, most bacterial taxa identified in the present investigation include mesophilic microorganisms. More specifically, the family Methylobacteriaceae includes mesophilic bacteria with optimum growth temperature of 30 °C [51,52]. Most species within the genus *Sphingomonas* grow in a temperature range from 15 to 35 °C, with optimum at 25–28 °C, although some other species in the same genus can grow at higher temperatures (around 40 °C) [53]. Members of the genus *Propionibacterium* grow in the range from 30 to 37 °C [54], whereas *Bacillus* spp. are generally characterized by optimum growth temperatures ranging between 25 and 40 °C [47]. This evidence might explain the growth of these bacteria during RTD, OD, and FD, as seems to be suggested by the results of viable plate counting.

In terms of mycobiota, a core of 13 genera was stably detected in the samples of dried sea fennel spices, including ubiquitous (*Aureobasidium*) and phytopathogenic (*Cladosporium*) molds and unconventional (*Sporobolomyces, Protomyces, Filobasidium, Kondoa*), plant-associated (*Symmetrospora, Vishniacozyma*), phytopathogenic (*Protomyces*, *Thelebolus*), ubiquitous (*Cryptococcus*), mycoparasitic (*Cystobasidium*), or food-associated yeasts (*Rhodotorula*). Other genera were detected in a few samples, namely *Alternaria, Tilletiopsis*, *Debaryomyces*, *Penicillium*, and *Taphrina*.

A few genera identified in this study, like *Cladosporium* [55] and *Cryptococcus* [56], include human pathogens. *Rhodotorula* and *Penicillium* have previously been identified as contaminants in herbs and spices [57]. The genus *Rhodotorula* includes yeast species that are capable of biosynthesizing lipids, carotenoids, and enzymes [58], whereas members of the genus *Penicillium* are capable of producing mycotoxins, such as *Penicillium verrucosum* and *Penicillium nordicum*, both producing ochratoxin A [59], a mycotoxin characterized by carcinogenic, immunotoxic, and nephrotoxic properties [60].

*Symmetrospora,* the most frequently detected yeast in the samples analyzed in this study, is characterized by optimum growth temperature in the range from 20 to 25 °C [61]; a previous investigation showed a decrease in these microorganisms as temperature increased during drying of tobacco leaves [62]. This evidence agrees well with the results of viable plate counting, which showed a reduction in the yeast population when OD and MD were applied to sea fennel leaves. Similarly, for the mold *Cladosporium,* the maximum growth temperature is 25 °C, while temperatures as high as 45 °C inhibit the growth of this microorganism [63]. Finally, the genus *Thelebolus* includes molds characterized by optimal growth temperature ranging from 4 to 15 °C, but some strains can grow at higher temperatures up to 45 °C [64].

Regarding the CIELab color parameters of dried sea fennel, the data collected in this study for OD, MD, and FD agreed with the data reported in previous studies. In more detail, the detection of the highest values for L* in the freeze-dried sea fennel samples was in line with the data collected by Renna et al. [25] and Giungato et al. [26]; furthermore, the increase in C in the dried samples agreed well with the results reported by Renna et al. [25]. Finally, the lowest reduction in the hue angle calculated for the samples subjected to MD and FD was again in line with the findings of Renna et al. [25], suggesting that these two drying methods generated a color that could potentially be perceived as more like fresh sea fennel [25].

When evaluating the effect of the drying methods on VOCs, a decrease in the content of some aroma compounds was observed in the dried samples compared to fresh sea fennel, with the effect being strictly dependent on the specific drying method adopted for most compounds. Limonene, gamma-terpinene, cymene, and sabinene, which occurred at the highest levels in fresh sea fennel, were drastically reduced after drying. The most important changes were observed in the oven-dried and freeze-dried sea fennel samples, where a drastic reduction in terpene hydrocarbons and an increase in some compounds, such as alcohols and esters, were observed. A more moderate reduction was observed in the sea fennel samples subjected to MD, while RTD had the least effect on the aroma pattern.

Díaz-Maroto et al. [65] also found that air drying of parsley at ambient temperature resulted in a small loss of volatile compounds compared to fresh herb, whereas oven drying at 45 °C and freeze drying caused a decrease in the concentrations of most volatile components, especially those with the greatest impact on parsley aroma. Furthermore, another research study carried out on dill revealed no differences in the volatile profile of freeze-dried and oven-dried samples [66]. Moreover, a moderate increase in the amount of some volatile compounds, such as aldehydes, alcohols, and esters, was observed in dried sea fennel, as a feasible consequence of the degradation of glycosylated forms, dehydration reactions, oxidation reactions, esterification reactions, or the release of compounds due to the rupture of cell walls, as previously suggested in other research studies [67,68,69]. Moreover, Turek and Stintzing [70] reported the formation of secondary aroma compounds, such as alcohols, aldehydes, peroxides, and ketones, during the drying process of essential oils, which substantially altered the volatile aroma profile of the final product. Nevertheless, the secondary products detected in our dried samples constituted a very low percentage of the total volatile content in the dried products.

The sensory analysis carried out by a panel of trained assessors allowed the qualitative and quantitative evaluation of key sensory parameters of dried sea fennel spices. As expected, odors and flavors typically associated with sea fennel, such as herbal and kerosene-like notes, were intensely perceived [17]. Although the application of different dehydration methods has previously been supposed to lead to changes in the volatilome of herbs, with consequent modification of their sensory parameters [71], no dramatic differences were observed when comparing the four drying methods investigated in this study. However, slight differences emerged as a more intense perception of herbal notes in the freeze-dried samples, or a higher intensity of earthy odor and tobacco flavor in the oven-dried samples, which agreed with the data previously reported by Renna and Gonnella [24]. Regarding overall appreciation, the microwave-dried samples received the highest scores, which was again in agreement with the data collected by Renna et al. [25], by comparing dried sea fennel spices obtained via air drying (at 45, 60, and 75 °C), microwave drying, freeze drying, and a combination of the latter two. Color, volatilome, and sensory traits are very important parameters in the study and production of dried spices. In this work, we compared four different drying methods, choosing for each one the most promising set of parameters according to the literature. In more detail, we select 45 °C as the temperature for the oven drying, considering it was reported to determine the lowest decay in the odor and color parameters with respect to higher temperatures, while ensuring a_w_ values < 0.6 [25].

## 4. Materials and Methods

### 4.1. Supply, Processing, and Moisture Content Determination of Sea Fennel

In November 2020, two batches of fresh sea fennel sprouts (each weighing ~4.5 Kg) were kindly provided by a local producer of sea fennel crop (Rinci S.r.l., Castelfidardo, Ancona, Italy). They were transported to the laboratory under refrigerated conditions (4 ± 2 °C), washed in an aqueous hypochlorite solution (60 mg L^−1^), rinsed with tap water, drained in an industrial stainless-steel strainer, gently dried with paper towels, and stored in plastic bags at 4 °C until use.

Prior to drying, for each batch, young sea fennel leaves were manually separated from stems and chopped with flame-sterilized steel scissors into small pieces of about 1.5–2 cm in length to facilitate water evaporation. For each batch, four aliquots (each weighing 50 g) of chopped sea fennel leaves were dried in a benchtop lab oven (ISCO, Milan, Italy) at 105 °C for 24 h [72] for the determination of the moisture content (wet basis, wb); the results were expressed as the mean values of four replicates per batch ± standard deviation.

### 4.2. Sea Fennel Drying

Aliquots of chopped sea fennel leaves from the two batches were dried in parallel using four drying methods, namely room-temperature drying (RTD), oven drying (OD), microwave drying (MD), and freeze drying (FD). For each batch and drying method, three replicates, each consisting of 330 g of chopped sea fennel leaves, were dried.

**Figure 6:**
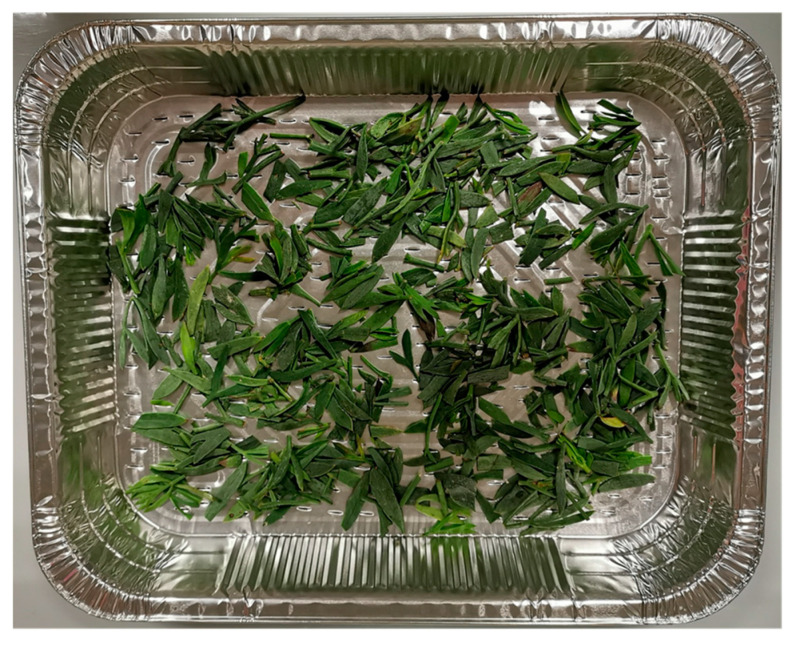
Chopped sea fennel leaves uniformly distributed on 20 × 30 cm aluminum trays, which were previously perforated at the bottom to prevent water stagnation.

In the RTD and OD trials, aliquots (55 g) of chopped sea fennel leaves were uniformly distributed on 20 × 30 cm aluminum trays, which had previously been perforated at the bottom to prevent water stagnation (Figure 6). The RTD trials were performed using a temperature-controlled chamber at 18 ± 2 °C, whereas the OD trials were carried out using a Heraeus function line B12/UB12 incubator (Thermo Fisher Scientific, Waltham, MA, USA) at 45 °C. In both RTD and OD trials, aluminum trays were weighed immediately before drying and at regular intervals during drying until weight stabilization.

**Figure 7:**
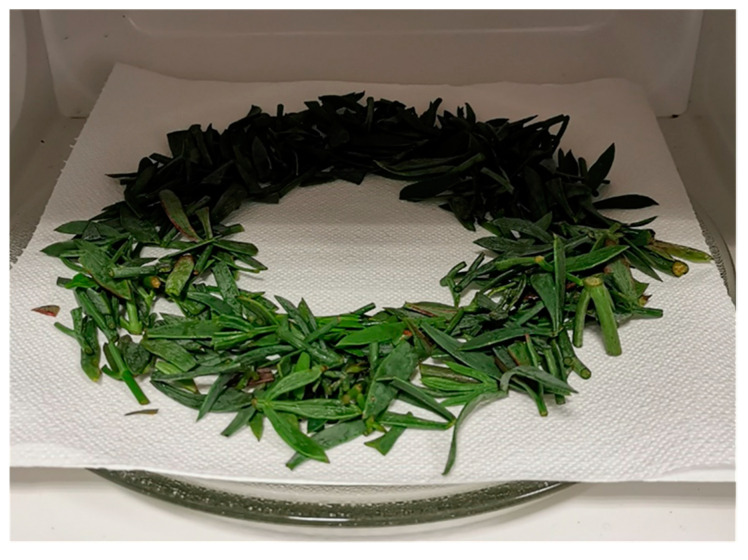
Aliquots (50 g) of chopped sea fennel leaves distributed circularly on the microwave plate on a paper towel.

The MD trials were performed using a WP 820 microwave oven (Amstrad, Brentwood, Essex, UK); aliquots (50 g) of chopped sea fennel leaves, distributed circularly on a paper towel on the microwave plate (Figure 7), were subjected to a treatment of decreasing energy output as suggested by Renna et al. [25], with slight modifications, as follows: (i) 800 W for 2 min; (ii) 450 W for 3 min; and (iii) 180 W for 10 (batch 1) or 3 (batch 2) minutes, until a constant weight was reached. To this end, sea fennel was weighed at regular intervals of 1 min.

Finally, the FD trials were carried out using a VirTis Wizard 2.0 freeze dryer (SP scientific, Warminster, PA, USA). Aliquots (80 g) of chopped sea fennel leaves were placed on aluminum trays to obtain a 2 mm layer and subjected to 24 h freeze drying.

### 4.3. Weight Loss Calculation and a_w_ Measurement

For each drying trial and batch, three replicates were individually weighed before and after drying using a precision scale WTB 2000 (Radwag, Radom, Poland); weight loss (%), referring to the initial weight of fresh sea fennel [25,26], was calculated as follows:

$$\frac{W_{i}-W_{f}\;}{W_{i}}\times 100$$

where

*W_i_* = initial weight;

*W_f_* = final weight.

The results were expressed as the mean of three replicates per batch ± standard deviation.

Aliquots of chopped fresh or dried sea fennel leaves were subjected to a_w_ measurement using an Aqualab 4TE apparatus (Meter Group, Pullman, WA, USA), in accordance with the standard ISO 18787:2017 method [73].

### 4.4. Viable Plate Counting

Aliquots (10 g) of chopped fresh and dried sea fennel leaves were added to 90 mL of 0.1% (w v^−1^) sterile peptone water and homogenized using a Stomacher 400 Circulator apparatus (International PBI, Milan, Italy) for 2 min at 230 rpm. The homogenates were ten-fold serially diluted in the same diluent and subjected to enumeration of (i) Enterobacteriaceae on Violet Red Bile Glucose Agar (VRBGA) (VWR, Radnor, PA, USA) incubated at 37 °C for 24 h; (ii) yeasts and molds on Rose Bengal Chloramphenicol agar (VWR) incubated at 25 °C for 5 days; and (iii) mesophilic aerobic bacteria and spore-forming bacteria on Plate Count Agar (PCA) (VWR) incubated at 30 °C for 72 h. For the enumeration of spore formers, prior to analysis, each homogenate was subjected to heat treatment at 80 °C for 10 min, followed by cooling in iced water, to inactivate vegetative cells. The results of viable plate counting were expressed as mean Log CFU g^−1^ of three replicates per batch ± standard deviation.

### 4.5. Metataxonomic Analysis

For each batch, aliquots (1 mL) of the homogenates of the three replicates, prepared as described in Section 4.4, were mixed for the preparation of bulk cells. The mixtures were centrifuged at 14,000 rpm for 10 min, and the resulting cell pellets were subjected to total DNA extraction using the E.Z.N.A. soil DNA kit (Omega bio-tek, Norcross, GA, USA) following the manufacturer’s instructions.

Microbiota and mycobiota were analyzed through the amplification of the bacterial 16S rRNA (V3–V4 region) [18] and fungal 26S rRNA genes (D1 domain) [74], respectively. Paired-end (2 × 250 bp) sequencing was performed using a MiSeq instrument (Illumina, San Diego, CA, USA), and QIIME2 software version 2022.2.0 [75] was used for data analysis by following the quality-filtering step of the dada2 denoise-paired plug in [76] to obtain the amplicon sequence variants (ASVs). The taxonomy assignment of ASVs was obtained using the Greengenes 16S rRNA database for bacteria, whereas the in-house database from Mota-Gutierrez et al. [77] was used for fungal ASVs. The sequences of each ASV were checked using the Basic Local Alignment Search Tool (BLAST), and the ASV tables were rarefied at the lowest number of sequence/sample to display the highest taxonomic resolution.

### 4.6. Color Assessment

The colorimetric profile of fresh and dried sea fennel was defined using a Chroma Meter CR-200 (Minolta, Japan) to determine lightness (L), redness–greenness (a*: + red; − green), and yellowness–blueness (b*: + yellow; − blue) coordinates according to the CIELab color space system with a D65 light source. Moreover, the hue angle (h°) was calculated using the formula h° = 180 + arctg (b*/a*) [78], and the chroma (C) was calculated using the formula C = [(a*^2^ + b*^2^)]^1/2^. For color assessment, whole fresh leaves were bound together to create a homogeneous surface, while the dried samples were ground to powder. The instrument was calibrated using standard white coordinates, and the colorimetric measurements were performed in triplicate. The results were expressed as the mean of three replicates per batch ± standard deviation.

### 4.7. Determination of Volatile Compounds via Headspace/Solid Phase Microextraction–Gas Chromatography/Mass Spectrometry (HS/SPME-GC/MS)

The volatile compounds were analyzed according to Reale et al. [79] with some modifications. In detail, aliquots (2 g) of chopped fresh and dried sea fennel leaves were placed in a 20 mL headspace vial (Gerstel GmbH & Co., Mülheim, Germany), and 5 µL of 4-methyl-2 pentanol (internal standard, 100 mg L^−1^ standard solution) was added. The samples were subjected to equilibration at 40 °C for 2 min at 250 rpm using a Gerstel MPS2 automatic sampling system (Gerstel GmbH & Co., Mülheim, Germany). The analysis was performed using a GC/MS system (Agilent 7890/5975 Inert, Agilent, Santa Clara, CA, USA) with helium as the carrier gas (1 mL min^−1^). A coated divinylbenzene/carboxen/polydimethylsiloxane (DVB/CAR/PDMS) fiber (Sigma Aldrich S.r.l., Milan, Italy) was exposed to each sample headspace for 15 min, while maintaining the sample temperature at 40 °C. The fiber was desorbed for 5 min at 240 °C in the injection unit in the split mode (split ratio 50:1). The separation was accomplished in a capillary column (HP-Innowax, Agilent Technologies, USA) (30 m × 0.25 mm i.d. × 0.50 μm film thickness). The GC oven’s temperature program started at 35 °C for 5 min, was then ramped to 150 °C at 5 °C min^−1^ and to 240 °C at 15 °C min^−1^, and maintained at the final temperature for 1 min. The mass spectrometer operated with an ion source of 230 °C, a quadrupole temperature of 150 °C, and 70 eV electron energy, and acquired in the TIC mode from m z^−1^ 30 to 350 uma. Identification of volatile compounds was carried out by comparing the mass spectra with the Wiley library (Wiley7, NIST 05). The results were expressed as RAP = relative peak area (peak area of compound/peak area of internal standard) × 100 (RAP ± SD). Blank experiments were conducted in two different modalities (blank of the fiber and blank of the empty vial) and analyzed after every 4 analyses. All analyses were performed in duplicate.

### 4.8. Sensory Analysis

The samples of dried sea fennel spices were subjected to sensory analysis by 10 trained non-smoking panelists, with half being male and half being female, and aged between 26 and 50, following the procedure previously described by Maoloni et al. [17] with slight modifications. Briefly, preliminary training sessions were carried out to select the most suitable attributes for dried sea fennel. The samples were coded with three-digit random numbers, and aliquots (1 g) were presented to the panel in white plastic cups at room temperature. The sensory analysis was divided into 6 sessions, each testing 4 samples; it was performed in individual booths equipped with coffee beans for olfactory cleansing and still bottled water for oral rinsing before and between the evaluations. The panelists assigned a score between 1 and 9, with 1 representing the lowest and 9 being the highest intensity, to seven odor and flavor descriptors (herbal, spicy, kerosene-like, earthy, tobacco, celery, and fresh) and four taste descriptors (sour, bitter, salty, and sweet). The overall liking was also evaluated using a 9-point hedonic scale, with 1 representing the lowest (extreme dislike) and 9 representing the highest (extreme like) degree of liking [80]. The results were expressed as the mean of three replicates per batch.

### 4.9. Statistical Analysis

One-way analysis of variance (ANOVA) was performed to detect significant differences between the four drying methods assayed within each batch and between the same drying method applied to the two different collection batches. The Tukey–Kramer honest significant difference (HSD) test was used with the criterion of significance set at *p* ≤ 0.05 using JMP Version 11.0.0 software (SAS Institute Inc., Cary, NC, USA). A principal component analysis (PCA) was also performed using the Tanagra 1.4 software with the dataset of VOCs.

## 5. Conclusions

All drying methods were able to remove the moisture content to achieve a_w_ values <0.6 for all the samples, thus rendering them microbiologically stable during storage.

The importance of a proper cleaning operation aimed at reducing the microbial contamination of fresh plant is pointed out, especially when using drying technologies that are not able to reduce the microbial load, such as room-temperature drying, oven drying at low temperature, or freeze drying. The application of different power levels and/or a prolonged process could be investigated to better evaluate spore inactivation via microwave drying. Finally, the results of the panel test highlighted a greater appreciation for microwave-dried spices.

Further studies are needed to assess the effect of each drying method on bioactive compounds in sea fennel, which can provide a clearer overview of the potential of each drying method in the production of high-quality spices with possible positive effect on human health.

Our results suggest that microwave drying might be a more suitable method for storing sea fennel spices than oven and freeze drying. However, as the quality of microwave-dried products is affected by drying parameters, such as microwave power (W), drying time, initial moisture content of the product, and dielectric properties of the materials [81], further trials are needed to identify the optimal storage conditions for sea fennel.

## Figures and Tables

**Table 1:** Drying parameters, weight loss, and water activity of dried sea fennel for each treatment.

Drying Treatment	Duration of the Drying Method		Weight Loss (%)		a_w_	
	Batch 1	Batch 2	Batch 1	Batch 2	Batch 1	Batch 2
RTD	16 d	16 d	84.68 ± 0.26 ^b,A^	82.69 ± 0.34 ^a,B^	0.58 ± 0.09 ^a,A^	0.49 ± 0.02 ^a,A^
OD	4 d	4 d	85.73 ± 0.22 ^a,A^	82.94 ± 0.09 ^a,B^	0.21 ± 0.06 ^b,A^	0.28 ± 0.02 ^b,A^
MD	15 min	8 min	85.67 ± 0.26 ^a,A^	82.63 ± 0.66 ^a,B^	0.20 ± 0.03 ^b,B^	0.40 ± 0.06 ^a,A^
FD	24 h	24 h	85.34 ± 0.39 ^ab,A^	83.15 ± 0.50 ^a,B^	0.26 ± 0.05 ^b,A^	0.16 ± 0.04 ^c,B^

Weight loss and water activity are expressed as mean value ± standard deviation. Values labeled with different small letters in the same column are significantly different (*p* < 0.05), whereas, for each parameter, values labeled with different capital letters in the same row are significantly different between the two batches (*p* < 0.05). RTD: room-temperature drying; OD: oven drying; MD: microwave drying; FD: freeze drying.

**Table 2:** Microbial viable counts of mesophilic aerobic bacteria, spore-forming bacteria, Enterobacteriaceae, yeasts, and molds in fresh and dried sea fennel samples.

Drying Treatment	Mesophilic Aerobic Bacteria		Spore Formers		Enterobacteriaceae		Yeasts		Molds	
	Batch 1	Batch 2	Batch 1	Batch 2	Batch 1	Batch 2	Batch 1	Batch 2	Batch 1	Batch 2
Control	6.0 ± 0.1 ^b,A^	4.7 ± 0.2 ^b,B^	2.3 ± 0.2 ^c,A^	2.7 ± 0.2 ^bc,A^	4.6 ± 0.1 ^ab,A^	3.3 ± 0.4 ^bc,B^	4.9 ± 0.2 ^a,A^	3.6 ± 0.4 ^b,B^	3.8 ± 0.1 ^b,A^	3.5 ± 0.1 ^a,B^
RTD	6.3 ± 0.3 ^ab,A^	4.7 ± 0.2 ^b,B^	2.8 ± 0.3 ^bc,B^	3.4 ± 0.2 ^b,A^	4.4 ± 0.1 ^b,A^	2.9 ± 0.1 ^c,B^	4.6 ± 0.3 ^ab,A^	3.2 ± 0.4 ^b,B^	4.0 ± 0.2 ^ab,A^	4.0 ± 0.4 ^a,A^
OD	7.0 ± 0.5 ^a,A^	4.7 ± 0.6 ^b,B^	4.7 ± 0.2 ^a,A^	4.3 ± 0.6 ^a,A^	5.7 ± 1.0 ^a,A^	3.5 ± 0.1 ^b,B^	2.2 ± 0.2 ^c,A^	2.2 ± 0.2 ^c,A^	4.3 ± 0.2 ^a,A^	3.9 ± 0.3 ^a,A^
MD	2.9 ± 0.2 ^c,A^	2.4 ± 0.1 ^c,B^	2.8 ± 0.2 ^bc,A^	2.5 ± 0.1 ^c,A^	< 1.0 ^c,A^	<1.0 ^d,A^	<1.0 ^d,A^	<1.0 ^d,A^	<1.0 ^c,A^	<1.0 ^b,A^
FD	6.2 ± 0.1 ^b,A^	5.5 ± 0.1 ^a,B^	3.2 ± 0.0 ^b,A^	3.3 ± 0.1 ^bc,A^	5.6 ± 0.0 ^a,A^	4.2 ± 0.1 ^a,B^	4.1 ± 0.2 ^b,B^	4.4 ± 0.0 ^a,A^	4.0 ± 0.2 ^ab,A^	3.9 ± 0.1 ^a,A^

The results are expressed as mean Log CFU g^−1^ of three replicates ± standard deviation. Values labeled with different small letters in the same column are significantly different (*p* < 0.05), whereas, for each microbial group, values labeled with different capital letters in the same row are significantly different between the two batches (*p* < 0.05). Control: chopped fresh sea fennel leaves; RTD: room-temperature drying; OD: oven drying; MD: microwave drying; FD: freeze drying.

**Table 3:** Colorimetric profile of chopped sea fennel leaves subjected to different drying methods.

Drying Treatment	Color Parameter									
	L		a*		b*		h°		C	
	Batch 1	Batch 2	Batch 1	Batch 2	Batch 1	Batch 2	Batch 1	Batch 2	Batch 1	Batch 2
Control	38.05 ± 1.45 ^d,A^	39.19 ± 1.29 ^c,A^	−8.31 ± 1.21 ^b,B^	−7.27 ± 0.57 ^a,A^	10.08 ± 1.46 ^d,A^	8.44 ± 0.84 ^d,B^	129.51 ± 1.28 ^a,B^	130.77 ± 0.81 ^a,A^	13.07 ± 1.88 ^d,A^	11.14 ± 1.00 ^d,B^
RTD	55.34 ± 0.57 ^b,B^	57.17 ± 0.94 ^a,A^	−12.11 ± 0.24 ^c,A^	−14.50 ± 0.19 ^d,B^	31.84 ± 0.35 ^b,B^	34.46 ± 0.50 ^a,A^	110.83 ± 0.23 ^c,B^	112.82 ± 0.14 ^c,A^	34.07 ± 0.41 ^b,B^	37.39 ± 0.52 ^a,A^
OD	49.30 ± 2.21 ^c,A^	50.38 ± 1.75 ^b,A^	−6.88 ± 0.50 ^a,A^	−9.52 ± 0.33 ^b,B^	26.11 ± 0.68 ^c,B^	28.44 ± 0.79 ^c,A^	104.75 ± 0.79 ^d,B^	108.51 ± 0.32 ^e,A^	27.00 ± 0.77 ^c,B^	29.99 ± 0.84 ^c,A^
MD	51.13 ± 1.47 ^c,A^	50.19 ± 0.83 ^b,A^	−13.40 ± 0.70 ^d,A^	−12.68 ± 1.71 ^c,A^	33.92 ± 0.76 ^a,A^	33.49 ± 0.49 ^b,A^	111.54 ± 0.65 ^c,A^	110.71 ± 2.63 ^d,A^	36.47 ± 0.95 ^a,A^	35.84 ± 0.70 ^b,A^
FD	60.24 ± 0.59 ^a,A^	57.99 ± 0.94 ^a,B^	−17.08 ± 0.26 ^e,A^	−17.95 ± 0.43 ^e,B^	32.57 ± 0.45 ^b,B^	33.41 ± 0.67 ^b,A^	117.67 ± 0.08 ^b,B^	118.24 ± 0.21 ^b,A^	36.77 ± 0.52 ^a,B^	37.92 ± 0.78 ^a,A^

CIELab color parameters: L, lightness; a*, redness–greenness; b*, yellowness–blueness; h°, hue angle; C, chroma. Results are expressed as mean value of three replicates ± standard deviation. Values labeled with different small letters in the same column are significantly different (*p* < 0.05), whereas, for each parameter, values labeled with different capital letters in the same row are significantly different between the two batches (*p* < 0.05). Control: chopped fresh sea fennel leaves; RTD: room-temperature drying; OD: oven drying; MD: microwave drying; FD: freeze drying.

**Table 4:** Volatile organic compounds (VOCs) identified in fresh sea fennel (C, control) and in samples of sea fennel spices obtained via room-temperature drying (RTD), oven drying (OD), freeze drying (FD), and microwave drying (MD).

		Drying Treatment									
RI	Compounds	C		RTD		OD		FD		**MD**	
		Batch 1	Batch 2	Batch 1	Batch 2	Batch 1	Batch 2	Batch 1	**Batch 2**	**Batch 1**	**Batch 2**
	**Aldehydes**										
**727**	acetaldehyde	19.92 ± 0.02 ^a,^*	23.68 ± 0.19 ^B,^*	10.79 ± 0.49 ^c,^*	17.12 ± 1.17 ^C,^*	2.27 ± 0.17 ^d^	2.61 ± 0.09 ^D^	14.78 ± 0.21 ^b,^*	29.35 ± 0.72 ^A,^*	nd ^e^	nd ^E^
**927**	3-methylbutanal	4.21 ± 0.23 ^a,^*	6.60 ± 0.43 ^A,^*	nd ^b^	nd ^C^	nd ^b^	nd ^C^	3.86 ± 0.42 ^a^	4.40 ± 0.04 ^B^	4.02 ± 0.06 ^a^	3.89 ± 0.38 ^B^
**1186**	heptanal	nd ^d^	nd ^C^	nd ^d^	nd ^C^	3.37 ± 0.26 ^b,^*	2.08 ± 0.18 ^B,^*	4.89 ± 0.53 ^a,^*	3.09 ± 0.20 ^A,^*	1.97 ± 0.05 ^c,^*	2.70 ± 0.01 ^A,^*
**1220**	2-hexenal	nd ^b^	nd ^B^	nd ^b^	nd ^B^	nd ^b^	nd ^B^	3.64 ± 0.15 ^a,^*	4.29 ± 0.15 ^A,^*	nd ^b^	nd ^B^
**1316**	octanal	nd ^c^	nd ^C^	nd ^c^	nd ^C^	7.95 ± 0.34 ^b,^*	5.27 ± 0.19 ^B,^*	9.67 ± 0.82 ^b,^*	1.78 ± 0.14 ^BC,^*	31.04 ± 1.67 ^a^	34.40 ± 2.16 ^A^
**1463**	2-furfural	nd ^b^	nd ^B^	nd ^b^	nd ^B^	nd ^b^	nd ^B^	nd ^b^	nd ^B^	37.94 ± 0.45 ^a^	37.64 ± 2.34 ^A^
**1488**	2,4-heptadienal	nd ^b^	nd ^B^	6.92 ± 0.38 ^a,^*	4.35 ± 0.39 ^A,^*	nd ^b^	nd ^B^	nd ^b^	nd ^B^	nd ^b^	nd ^B^
**1598**	5-methylfurfural	nd ^b^	nd ^B^	nd ^b^	nd ^B^	nd ^b^	nd ^B^	nd ^b^	nd ^B^	14.77 ± 0.66 ^a^	14.78 ± 0.18 ^A^
**1631**	2-decenal	nd ^b^	nd ^B^	nd ^b^	nd ^B^	nd ^b^	nd ^B^	nd ^b^	nd ^B^	4.36 ± 0.23 ^a,^*	6.30 ± 0.41 ^A,^*
	sub total	24.14 ± 0.25 ^c,^*	30.29 ± 0.62 ^C,^*	17.71 ± 0.87 ^d^	21.47 ± 1.56 ^C^	13.59 ± 0.43 ^d,^*	9.97 ± 0.46 ^D,^*	36.84 ± 0.99 ^b,^*	42.92 ± 0.87 ^B,^*	94.10 ± 2.57 ^a^	99.70 ± 5.47 ^A^
	**Alcohols**										
**955**	ethyl alcohol	309.20 ± 1.97 ^d^	352.03 ± 14.75 ^C^	1365.43 ± 112.06 ^b^	1230.96 ± 69.99 ^B^	1831.70 ± 5.97 ^a^	1825.35 ± 114.70 ^A^	1152.54 ± 12.38 ^c^	1148.68 ± 34.91 ^B^	1038.23 ± 6.35 ^c^	1038.73 ± 10.23 ^B^
**1634**	2-furanmethanol	nd ^b^	nd ^B^	nd ^b^	nd ^B^	nd ^b^	nd ^B^	nd ^b^	nd ^B^	24.40 ± 0.49 ^a^	24.40 ± 1.09 ^A^
**1852**	phenethyl alcohol	nd ^b^	nd ^B^	nd ^b^	nd ^B^	2.37 ± 0.01 ^a,^*	2.15 ± 0.05 ^A,^*	nd ^b^	nd ^B^	nd ^b^	nd ^B^
	sub total	309.20 ± 1.97 ^d^	352.03 ± 14.75 ^C^	1365.43 ± 112.06 ^b^	1230.96 ± 69.78 ^B^	1834.07 ± 5.98 ^a^	1827.51 ± 114.76 ^A^	1152.54 ± 12.38 ^c^	1148.68 ± 34.91 ^B^	1062.62 ± 6.84 ^c^	1063.13 ± 11.32 ^B^
	**Esters**										
**1222**	isobutyl 2-butanoate	nd ^b^	nd ^B^	nd ^b^	nd ^B^	nd ^b^	nd ^B^	2.40 ± 0.06 ^a^	2.32 ± 0.06 ^A^	nd ^b^	nd ^B^
**1339**	ethyl heptanoate	nd ^c^	nd ^C^	nd ^c^	nd ^C^	2.54 ± 0.03 ^b,^*	1.98 ± 0.08 ^B,^*	3.47 ± 0.29 ^a^	3.29 ± 0.03 ^A^	nd ^c^	nd ^C^
**1466**	ethyl octanoate	nd ^d^	nd ^C^	71.29 ± 4.67 ^a^	54.61 ± 4.75 ^A^	55.66 ± 0.81 ^b^	56.84 ± 1.69 ^A^	10.29 ± 0.07 ^c,^*	30.20 ± 0.32 ^B,^*	nd ^d^	nd ^C^
**1530**	ethyl nonanoate	nd ^b^	nd ^B^	11.47 ± 0.77 ^a^	11.93 ± 0.30 ^A^	nd ^b^	nd ^B^	nd ^b^	nd ^B^	nd ^b^	nd ^B^
**907**	ethyl acetate	nd ^d^	nd ^D^	6.82 ± 0.92 ^b^	6.17 ± 0.68 ^B^	4.05 ± 0.27 ^c^	3.68 ± 0.37 ^C^	10.72 ± 0.30 ^a,^*	12.04 ± 0.33 ^A,^*	nd ^d^	nd ^D^
**1607**	ethyl decanoate	nd ^c^	nd ^D^	11.00 ± 1.30 ^a^	8.86 ± 0.79 ^A^	5.72 ± 0.37 ^b,^*	4.29 ± 0.06 ^B,^*	1.44 ± 0.07 ^c,^*	2.26 ± 0.01 ^C,^*	nd ^c^	nd ^D^
	sub total	nd ^d^	nd ^D^	100.58 ± 7.66 ^a^	81.57 ± 6.53 ^A^	67.97 ± 0.13 ^b^	66.79 ± 2.20 ^B^	28.31 ± 0.23 ^c,^*	50.10 ± 0.55 ^C,^*	nd ^d^	nd ^D^
	**Acids**										
**1477**	acetic acid	1.74 ± 0.06 ^d^	1.98 ± 0.06 ^D^	10.03 ± 0.94 ^c,^*	6.13 ± 0.64 ^C,^*	18.97 ± 0.35 ^b^	15.11 ± 1.37 ^B^	nd ^d^	nd ^D^	24.51 ± 0.08 ^a^	25.80 ± 1.29 ^A^
	**Terpenes ** **hydrocarbons**										
**1010**	alpha pinene	236.04 ± 1.59 ^a,^*	207.42 ± 5.69 ^A,^*	195.93 ± 21.05 ^b^	154.28 ± 8.04 ^B^	28.07 ± 0.68 ^d,^*	33.55 ± 1.19 ^E,^*	62.14 ± 6.20 ^d^	54.57 ± 5.05 ^D^	110.46 ± 3.35 ^c^	102.53 ± 0.92 ^C^
**1000**	alpha thujene	120.29 ± 4.18 ^a^	112.14 ± 0.61 ^A^	110.73 ± 12.64 ^a^	95.97 ± 6.91 ^B^	18.10 ± 0.99 ^c,^*	23.49 ± 0.39 ^D,^*	36.85 ± 0.36 ^bc^	34.29 ± 4.09 ^D^	52.45 ± 5.56 ^b^	53.37 ± 3.94 ^C^
**1111**	camphene	8.95 ± 0.19 ^b,^*	6.59 ± 0.19 ^B,^*	11.80 ± 0.97 ^a^	8.56 ± 0.55 ^A^	nd ^d^	nd ^E^	3.90 ± 0.74 ^c^	2.20 ± 0.14 ^D^	4.14 ± 0.25 ^c^	3.85 ± 0.04 ^C^
**1125**	beta pinene	15.88 ± 0.27 ^a,^*	11.51 ± 0.14 ^A,^*	14.31 ± 1.21 ^a^	11.40 ± 0.83 ^A^	5.61 ± 0.62 ^b^	3.68 ± 0.33 ^C^	6.83 ± 0.35 ^b^	6.12 ± 0.49 ^B^	7.44 ± 0.08 ^b^	6.90 ± 0.57 ^B^
**1145**	sabinene	2039.22 ± 65.45 ^a^	1970.01 ± 35.71 ^A^	1049.55 ± 12.70 ^b^	1135.26 ± 40.35 ^B^	120.73 ± 8.51 ^e,^*	185.07 ± 2.95 ^D,^*	403.63 ± 40.95 ^d^	360.31 ± 35.97 ^CD^	584.29 ± 31.17 ^c^	457.71 ± 99.12 ^C^
**1163**	delta 3-carene	10.71 ± 0.54 ^a^	10.23 ± 0.18 ^A^	7.45 ± 1.02 ^b^	6.61 ± 0.51 ^B^	6.17 ± 0.17 ^b^	6.20 ± 0.24 ^B^	nd ^d^	nd ^D^	3.57 ± 0.44 ^c^	2.21 ± 0.11 ^C^
**1173**	alpha phellandrene	5.38 ± 0.38 ^a^	5.87 ± 0.47 ^A^	5.98 ± 0.20 ^a^	3.94 ± 1.17 ^AB^	nd ^c^	nd ^C^	nd ^c^	nd ^C^	2.70 ± 0.00 ^b,^*	3.17 ± 0.06 ^B,^*
**1176**	beta myrcene	445.43 ± 22.02 ^a,^*	313.19 ± 4.80 ^A,^*	268.49 ± 22.77 ^b^	251.98 ± 13.58 ^B^	32.30 ± 3.09 ^d,^*	79.29 ± 2.90 ^D,^*	60.83 ± 0.34 ^d^	62.16 ± 5.12 ^D^	175.68 ± 12.91 ^c,^*	116.34 ± 9.70 ^C,^*
**1182**	alpha terpinene	30.59 ± 0.41 ^a^	20.64 ± 13.81 ^A^	19.61 ± 1.61 ^ab^	20.24 ± 0.38 ^A^	nd ^b^	nd ^A^	9.24 ± 0.25 ^ab,^*	10.48 ± 0.00 ^A,^*	2.83 ± 0.00 ^b^	2.92 ± 0.04 ^A^
**1189**	limonene	25,364.65 ± 165.02 ^a^	26,686.91 ± 630.78 ^A^	14,507.89 ± 1602.44 ^b^	14,372.74 ± 753.00 ^B^	2726.30 ± 79.08 ^d^	3470.77 ± 320.34 ^D^	2937.24 ± 196.19 ^d^	2676.22 ± 130.93 ^D^	8249.83 ± 594.86 ^c^	6861.19 ± 683.87 ^C^
**1199**	beta phellandrene	313.19 ± 4.56 ^a,^*	242.41 ± 3.48 ^A,^*	204.79 ± 15.74 ^b^	222.03 ± 5.80 ^B^	58.77 ± 3.79 ^d^	64.17 ± 1.29 ^D^	33.98 ± 0.64 ^d,^*	52.54 ± 1.97 ^D,^*	103.77 ± 0.63 ^c^	95.32 ± 6.07 ^C^
**1249**	gamma terpinene	27,282.75 ± 518.82 ^a,^*	15,454.02 ± 634.48 ^A,^*	12,991.31 ± 444.36 ^b^	12,214.91 ± 833.83 ^B^	1345.17 ± 52.52 ^d,^*	3434.37 ± 323.16 ^CD,^*	2277.27 ± 42.75 ^d^	2267.66 ± 63.71 ^D^	7042.27 ± 535.88 ^c^	4691.32 ± 735.31 ^C^
**1263**	trans ocimene	126.79 ± 0.68 ^cd,^*	385.84 ± 37.40 ^B,^*	617.80 ± 38.54 ^a^	781.77 ± 66.49 ^A^	54.93 ± 0.13 ^d,^*	372.28 ± 31.45 ^BC,^*	171.08 ± 1.72 ^c,^*	195.96 ± 3.16 ^C,^*	512.28 ± 38.53 ^b^	455.68 ± 54.16 ^B^
**1280**	cymene	10,888.52 ± 342.92 ^a,^*	15,862.95 ± 785.66 ^A,^*	2517.19 ± 175.31 ^b^	2047.97 ± 103.70 ^B^	636.99 ± 31.81 ^cd^	669.30 ± 35.31 ^BC^	566.86 ± 27.48 ^d^	553.56 ± 12.31 ^C^	1291.51 ± 112.59 ^c^	1312.95 ± 39.03 ^BC^
**1408**	1,3,8-p-menthatriene	nd ^b^	nd ^B^	nd ^b^	nd ^B^	nd ^b^	nd ^B^	6.10 ± 0.37 ^a^	5.76 ± 0.49 ^A^	nd ^b^	nd ^B^
**1374**	neo allocimene	5.71 ± 0.10 ^b,^*	4.24 ± 0.17 ^B,^*	6.89 ± 0.34 ^a^	6.00 ± 0.71 ^A^	3.33 ± 0.31 ^c^	3.73 ± 0.20 ^B^	3.43 ± 0.37 ^c^	2.82 ± 0.23 ^B^	6.71 ± 0.19 ^ab^	6.48 ± 0.48 ^A^
	sub total	66,894.10 ± 791.43 ^a^	61,294.00 ± 2152.58 ^A^	32,519.38 ± 2310.81 ^b^	31,333.64 ± 1835.86 ^B^	5036.71 ± 116.24 ^d,^*	8345.90 ± 646.44 ^CD,^*	6579.36 ± 316.81 ^d^	6284.66 ± 259.69 ^D^	18,149.96 ± 1336.26 ^c^	14,171.95 ± 1553.42 ^C^
	**Oxygenated terpenes**										
**1467**	cis limonene oxide	8.71 ± 0.40 ^a,^*	13.58 ± 0.34 ^A,^*	7.47 ± 1.04 ^ab^	10.09 ± 0.10 ^B^	nd ^c^	nd ^E^	8.49 ± 0.71 ^a^	7.42 ± 1.05 ^C^	5.10 ± 0.35 ^b^	4.65 ± 0.06 ^D^
**1473**	trans limonene oxide	56.31 ± 4.04 ^a,^*	136.87 ± 5.68 ^A,^*	nd ^b^	nd ^B^	nd ^b^	nd ^B^	4.82 ± 0.43 ^b^	4.48 ± 0.53 ^B^	2.30 ± 0.04 ^b^	1.74 ± 0.67 ^B^
**2163**	thymol	27.37 ± 0.45 ^a^	29.81 ± 1.04 ^A^	nd ^b^	nd ^B^	nd ^b^	nd ^B^	nd ^b^	nd ^B^	nd ^b^	nd ^B^
**2187**	carvacrol	23.14 ± 0.14 ^a,^*	10.84 ± 0.38 ^A,^*	nd ^b^	nd ^B^	nd ^b^	nd ^B^	nd ^b^	nd ^B^	nd ^b^	nd ^B^
**1563**	thymyl methyl ether	77.33 ± 1.36 ^a^	68.83 ± 3.53 ^A^	46.30 ± 1.43 ^b^	41.65 ± 3.79 ^B^	6.16 ± 0.23 ^e,^*	9.16 ± 0.73 ^D,^*	10.25 ± 0.52 ^d^	9.55 ± 0.64 ^D^	23.43 ± 0.84 ^c^	22.01 ± 0.03 ^C^
**1577**	isothymol methyl ether	10.74 ± 0.58 ^b,^*	14.15 ± 0.01 ^A,^*	8.27 ± 0.51 ^c,^*	12.02 ± 0.18 ^B,^*	1.47 ± 0.08 ^e,^*	4.13 ± 0.27 ^C,^*	3.75 ± 0.51 ^d^	3.85 ± 0.16 ^C^	13.79 ± 0.68 ^a^	12.88 ± 0.96 ^AB^
**1579**	carvacrol methyl ether	19.90 ± 0.05 ^a^	19.21 ± 0.48 ^A^	7.73 ± 0.99 ^b^	8.19 ± 0.14 ^B^	1.99 ± 0.08 ^d,^*	3.17 ± 0.02 ^CD,^*	2.66 ± 0.03 ^d^	2.74 ± 0.08 ^D^	5.31 ± 0.65 ^c^	4.47 ± 0.67 ^C^
	sub total	223.50 ± 5.05 ^a,^*	293.29 ± 11.48 ^A,^*	69.78 ± 3.97 ^b^	71.94 ± 4.20 ^B^	9.62 ± 0.39 ^e,^*	16.46 ± 0.44 ^D,^*	29.97 ± 2.19 ^d^	28.04 ± 2.47 ^CD^	49.93 ± 2.56 ^c^	45.74 ± 0.86 ^C^
	**Sesquiterpenes ** **hydrocarbons**										
**1576**	alpha bergamotene	nd ^b^	nd ^B^	10.28 ± 1.34 ^a^	9.66 ± 0.70 ^A^	nd ^b^	nd ^B^	nd ^b^	nd ^B^	nd ^b^	nd ^B^
**1780**	germacrene B	15.77 ± 0.16 ^a^	14.88 ± 0.55 ^A^	10.55 ± 1.26 ^b^	10.19 ± 0.56 ^B^	3.80 ± 0.36 ^c^	4.23 ± 0.29 ^C^	4.37 ± 0.21 ^c^	4.60 ± 0.12 ^C^	9.24 ± 0.26 ^b^	10.06 ± 0.18 ^B^
**1687**	zingiberene	3.14 ± 0.08 ^b,^*	2.74 ± 0.07 ^B,^*	2.93 ± 0.34 ^b^	2.05 ± 0.14 ^C^	nd ^c^	nd ^D^	nd ^c^	nd ^D^	6.28 ± 0.67 ^a^	6.43 ± 0.28 ^A^
**1648**	gamma elemene	15.90 ± 0.35 ^b,^*	17.91 ± 0.15 ^BC,^*	42.88 ± 1.67 ^a^	48.31 ± 0.66 ^A^	15.76 ± 0.94 ^b^	17.71 ± 0.05 ^C^	15.35 ± 0.07 ^b,^*	19.42 ± 0.56 ^B,^*	11.09 ± 0.40 ^c^	11.20 ± 0.00 ^D^
**1694**	bicyclogermacrene	7.19 ± 0.18 ^a^	6.93 ± 0.29 ^A^	7.99 ± 1.06 ^a^	6.87 ± 0.71 ^A^	nd ^c^	nd ^C^	2.93 ± 0.07 ^b^	2.83 ± 0.04 ^B^	nd ^c^	nd ^C^
**1473**	copaene	nd ^b^	nd ^B^	3.58 ± 0.31 ^a^	5.07 ± 0.49 ^A^	nd ^b^	nd ^B^	nd ^b^	Nd ^B^	nd ^b^	nd ^B^
**1557**	caryophyllene	12.98 ± 0.16 ^a,^*	8.28 ± 0.33 ^A,^*	10.86 ± 0.59 ^b,^*	8.53 ± 0.15 ^A,^*	3.06 ± 0.10 ^d^	4.34 ± 0.55 ^B^	3.22 ± 0.13 ^d^	2.84 ± 0.17 ^C^	5.40 ± 0.31 ^c^	5.00 ± 0.29 ^B^
**1538**	alpha longipinene	21.04 ± 0.05 ^a,^*	19.12 ± 0.13 ^A,^*	19.68 ± 1.60 ^a^	17.08 ± 0.56 ^B^	5.45 ± 0.34 ^b^	5.69 ± 0.14 ^C^	4.17 ± 0.03 ^b,^*	5.34 ± 0.32 ^C,^*	3.62 ± 0.18 ^b^	3.91 ± 0.35 ^D^
**1790**	cuparene	4.41 ± 0.31 ^a^	4.60 ± 0.25 ^A^	3.82 ± 0.52 ^a^	3.41 ± 0.06 ^B^	1.85 ± 0.05 ^b,^*	2.23 ± 0.05 ^C,^*	nd ^c^	nd ^D^	1.49 ± 0.02 ^b,^*	1.86 ± 0.02 ^C,^*
**2207**	elemicine	nd ^b^	nd ^B^	nd ^b^	nd ^B^	nd ^b^	nd ^B^	3.56 ± 0.36 ^a^	3.19 ± 0.17 ^A^	nd ^b^	nd ^B^
	sub total	80.43 ± 0.51 ^b,^*	74.46 ± 0.38 ^B,^*	112.58 ± 8.70 ^a^	111.18 ± 2.70 ^A^	29.93 ± 1.79 ^c^	34.19 ± 0.98 ^C^	33.60 ± 0.25 ^c,^*	38.23 ± 0.50 ^C,^*	37.12 ± 0.33 ^c^	38.45 ± 1.13 ^C^
	**Phenyl propanoids**										
**2327**	dillapiole	25.59 ± 0.32 ^c^	27.11 ± 0.44 ^D^	54.20 ± 7.84 ^b^	51.50 ± 0.22 ^B^	13.03 ± 0.68 ^c,^*	15.32 ± 0.32 ^E,^*	76.18 ± 7.92 ^a^	71.91 ± 1.22 ^A^	31.86 ± 2.84 ^c^	32.49 ± 0.08 ^C^

Values labeled with different small letters in the same row are significantly different (*p* < 0.05) in batch 1, and values labeled with different capital letters in the same row are significantly different (*p* < 0.05) in batch 2, whereas, for each drying method, values marked with * in the same row are significantly different among the two batches (*p* < 0.05). Abbreviations: nd, not detected; RI = retention index, identification via comparison with RI database. Results are expressed as RAP = relative peak area (peak area of compound/peak area of internal standard) × 100 (RAP ± SD). www.webbook.nist.gov (Accessed on 15 June 2023).

## Data Availability

Data are contained within the article and Supplementary Materials.

## Supplementary Materials

The following supporting information can be downloaded at https://www.mdpi.com/article/10.3390/molecules28207207/s1, Table S1: Bacterial amplicon sequencing variants (ASVs) detected (●) in the samples of dried sea fennel spices. For an explanation of the drying methods and batches used, see Table 1; Table S2: Fungal amplicon sequencing variants (ASVs) detected (●) in the dried sea fennel samples. For an explanation of the drying methods and batches used, see Table 1.
