# Supplementary material for: Impact of Different Drying Methods on the Microbiota, Volatilome, Color, and Sensory Traits of Sea Fennel (Crithmum maritimum L.) Leaves
Source: Molecules. 2023 Oct 21;28(20):7207. doi: 10.3390/molecules28207207 (PMC10609079; doi:10.3390/molecules28207207)
Supplement: Supplementary file 1 [file molecules-28-07207-s001.zip › molecules-2611241-supplementary.pdf]

Table S1. Bacterial amplicon sequencing variants (ASVs) detected (●) in the samples of dried sea fennel spices. For an explanation of the drying methods and batches used, see Table 1.

**Sample**	**Batch**	*Bacillus*	*Brachybacterium*	Caulobacteraceae	*Devosia*	*Erwinia*	Gaiellaceae	Methylobacteriaceae	Microbacteriaceae	*Propionibacterium*	*Pseudomonas*	Rhodobacteraceae	*Sphingomonas*
OD	1									●			
	2			●		●	●	●	●		●	●	
MD	1	●						●					●
	2							●					●
FD	1		●	●	●								
	2												●

Table S2. Fungal amplicon sequencing variants (ASVs) detected (●) in the dried sea fennel samples. For an explanation of the drying methods and batches used, see Table 1.

Drying treatment	Batch	*Alternaria*	*Aureobasidium*	*Bensingtonia*	*Cladosporium*	*Cryptococcus*	*Cystobasidium*	*Debaryomyces*	*Filobasidium*	*Kondoa*	*Penicillium*	*Protomyces*	*Rhodotorula*	*Sporobolomyces*	*Symmetrospora*	*Taphrina*	*Thelebolus*	*Tilletiopsis*	*Vishniacozyma*
RTD	1	●	●	●	●	●	●		●	●		●	●	●	●	●	●		●
	2	●		●	●	●	●		●	●	●			●	●		●	●	●
OD	1	●	●	●	●	●	●		●	●		●	●	●	●	●	●		●
	2	●	●	●	●		●		●	●		●	●	●	●	●	●		●
MD	1	●	●	●	●	●	●		●	●		●	●	●	●	●	●	●	●
	2	●	●	●	●	●	●		●	●		●	●	●	●	●	●	●	●
FD	1		●	●	●	●	●		●	●		●	●	●	●		●		●
	2			●	●			●				●		●	●				●
